# Mixed YOLOv3-LITE: A Lightweight Real-Time Object Detection Method

**DOI:** 10.3390/s20071861

**Published:** 2020-03-27

**Authors:** Haipeng Zhao, Yang Zhou, Long Zhang, Yangzhao Peng, Xiaofei Hu, Haojie Peng, Xinyue Cai

**Affiliations:** 1 The Institute of Geospatial Information, Strategic Support Force Information Engineering University, Zhengzhou 450001, China; haipengzhao@cumt.edu.cn (H.Z.); pengyangzhao@163.com (Y.P.); huxiaofeicn@163.com (X.H.); penghhjj@163.com (H.P.); xinyueCCC@163.com (X.C.); 2 Beijing Institute of Remote Sensing Information, Beijing 100192, China; jagger_3d@126.com

**Keywords:** object detection, computer vision, convolutional neural network, embedded system, real-time performance

## Abstract

Embedded and mobile smart devices face problems related to limited computing power and excessive power consumption. To address these problems, we propose Mixed YOLOv3-LITE, a lightweight real-time object detection network that can be used with non-graphics processing unit (GPU) and mobile devices. Based on YOLO-LITE as the backbone network, Mixed YOLOv3-LITE supplements residual block (ResBlocks) and parallel high-to-low resolution subnetworks, fully utilizes shallow network characteristics while increasing network depth, and uses a “shallow and narrow” convolution layer to build a detector, thereby achieving an optimal balance between detection precision and speed when used with non-GPU based computers and portable terminal devices. The experimental results obtained in this study reveal that the size of the proposed Mixed YOLOv3-LITE network model is 20.5 MB, which is 91.70%, 38.07%, and 74.25% smaller than YOLOv3, tiny-YOLOv3, and SlimYOLOv3-spp3-50, respectively. The mean average precision (mAP) achieved using the PASCAL VOC 2007 dataset is 48.25%, which is 14.48% higher than that of YOLO-LITE. When the VisDrone 2018-Det dataset is used, the mAP achieved with the Mixed YOLOv3-LITE network model is 28.50%, which is 18.50% and 2.70% higher than tiny-YOLOv3 and SlimYOLOv3-spp3-50, respectively. The results prove that Mixed YOLOv3-LITE can achieve higher efficiency and better performance on mobile terminals and other devices.

## 1. Introduction

Recently, object detection based on convolutional neural networks has been a popular research topic in the field of computer vision with a focus on object location and classification. Feature extraction and classification of original images can be conducted via multi-layer convolution operations, and the position of an object in an image can be predicted using boundary boxes, providing the capability of visual understanding. The results of these studies can be widely applied in facial recognition [1], attitude prediction [2], video surveillance, and a variety of other intelligent applications [3,4,5].

Currently, convolutional neural network structures are becoming deeper and more complex. Although such network structures can match or even exceed human vision in precision, they usually require huge amounts of computation power and involve ultra-high energy consumption. There has been significant development in fast object detection methods [6,7,8]; nevertheless, it is still inconvenient to implement convolutional neural network structures in non-graphics processing unit (GPU) or mobile devices. With the growth in the development of embedded and mobile intelligent devices with limited computing power and power consumption, such as small intelligent unmanned aerial vehicles (UAVs) and augmented reality glasses, lightweight and real-time network models [9,10] have become key areas of research on the use of convolutional neural network-based object detection technology in mobile applications.

Recently, some researchers have focused on improving detection precision by building more complex neural networks, such as the deep residual network (ResNet) [11], dense convolutional network (DenseNet) [12], dual-path network (DPN) [13], YOLOv3 [14], and high-resolution network (HRNet) [15]. Other researchers have constructed small and efficient lightweight neural networks by optimizing various structures, such as MobileNetV1 [16], MobileNetV2 [17], Tiny-YOLO [18], and YOLO-LITE [19]. End-to-end deep-learning object detection methods based on regression methods, such as the YOLO series [14,19,20,21,22,23] and single shot multi-box detector (SSD) series [24,25,26], have achieved real-time object detection with GPU-based computers while maintaining a relatively high average precision. However, because of the intensive computational requirements of such systems, it is difficult to achieve real-time and precise detection using non-GPU based computers and portable devices that have limited computing power.

To overcome these limitations, we propose in this study a lightweight object detection network, Mixed YOLOv3-LITE, that uses a shallow-layer, narrow-channel, and multi-scale feature-image parallel fusion structure. Mixed YOLOv3-LITE can ensure a certain accuracy rate and its characteristics of less computation than conventional methods and fast detection speed mean that it can be implemented in embedded and mobile devices for accurate and efficient object detection. The main contributions of this paper are as follows:

1. The proposed Mixed YOLOv3-LITE fuses deep and shallow features and output feature maps at different scales to maximize the utilization of the original features by incorporating ResBlocks and parallel structures.
2. The convolution layers of the Mixed YOLOv3-LITE detector are shallower and narrower, which reduce the amount of computation and the number of trainable parameters to speed up the operation of the network.
3. Mixed YOLOv3-LITE with fewer parameters—only about 5.089 million—is a lightweight real-time network that can be implemented on mobile terminals and other non-GPU based devices.

The remainder of this paper is organized as follows. Section 2 describes several complex networks with high precision and some efficient lightweight networks. Section 3 presents the proposed network model and describes its structure in detail. Section 4 describes the datasets, evaluation indicators, and experimental conditions adopted in the model evaluation process. It also describes comparative experiments carried out to assess the model and compares the experimental results with different datasets. Finally, Section 5 presents conclusions drawn from the results and future prospects.

## 2. Related Work

### 2.1. Complex Networks with High Precision

In this subsection, complex neural networks such as ResNet, YOLOv3, and HRNet are described in detail. We extracted their core structures for use in the experiments of this study.

#### 2.1.1. Deep Residual Network (ResNet), DenseNet, and Dual-Path Network (DPN)

ResNet was proposed by He et al. [11], who applied the concept of residual representation, which is commonly used in conventional computer vision, to construct a convolutional neural network (CNN) model. They also proposed a ResBlock structure, which adds a shortcut to the network. Their approach is effective to an extent in solving the problem of the precision of a model decreasing when the number of layers in a visual geometry group (VGG) [27] network increases.

DenseNet, proposed by Huang et al. [12], is a method in which each layer accepts feature mapping from all previous layers, thereby making the network thinner and more compact. This network has fewer parameters than ResNet, which strengthens the reuse of features and mitigates the problems of gradient vanishing and model degradation.

The dual-path network is a simple, efficient, and modular network proposed by Chen et al. [13]. This network uses dual-path topology in combination with the feature reuse in ResNet and new features exploration in DenseNet, to achieve common feature sharing, ensure flexibility, and explore new features.

#### 2.1.2. YOLOv3

**Figure 1:**
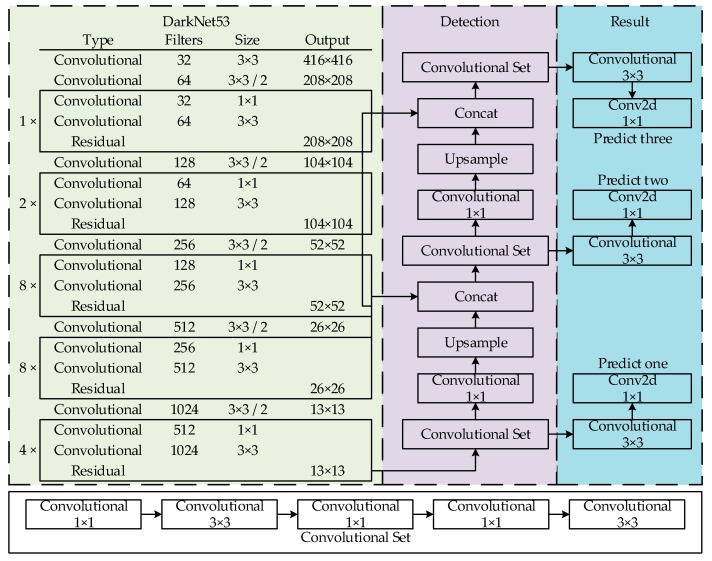
YOLOv3 network structure.

YOLOv3 [14] learns from a residual network structure to form a deeper network level. It uses multi-scale features for object detection and logistics instead of softmax object classification to improve the mean average precision (mAP) and detection of small objects. In the case of equal precision, the speed of YOLOv3 is three to four times greater than that of other models. Its network structure is illustrated in Figure 1.

#### 2.1.3. High-Resolution Network (HRNet)

HRNet proposed by Sun et al. [15] maintains a high-resolution representation by parallel subnetworks of high-resolution to low-resolution convolution and enhances high-resolution representation by repeatedly performing multi-scale fusion across parallel convolution. This network can maintain high-resolution representation rather than just recover high-resolution representation from low-resolution representation. The effectiveness of the method was demonstrated in pixel-level classification, region-level classification, and image-level classification.

### 2.2. Lightweight Networks

#### 2.2.1. MobileNetV1 and MobileNetV2

MobileNetV1 [16] and MobileNetV2 [17] are efficient models proposed by Google for mobile and embedded devices. MobileNetV1 is based on a streamlined structure. Its underlying innovation is the use of depthwise-separable convolutions (Xception) to build a lightweight-depth neural network that greatly reduces the number of parameters and the amount of computation. It also achieves a desirable balance between detection speed and precision by introducing the parameters α (width multiplier) and ρ (resolution multiplier). Based on deep separable convolution, MobileNetV2 uses the inverted residual and linear bottleneck structure to maintain the representation ability of the model.

#### 2.2.2. Tiny-YOLO and YOLO-LITE

Tiny-YOLO [18] is a lightweight implementation of the YOLO network. It can be used as an alternative structure for YOLOv2 or YOLOv3 in scenarios where the demand for precision is not high. Its detection speed is faster than that of the original network. However, in the case of non-GPU based devices, Tiny-YOLO still encounters difficulty meeting the requirements of real-time detection. YOLO-LITE [19] is a lightweight version of YOLOv2, which is faster than Tiny-YOLOv2 but with a lower average precision.

This section introduced complex networks such as ResNet, YOLOv3, and HRNet, and lightweight networks such as MobileNet and YOLO-LITE. Owing to the large amount of model parameters and computation, high requirements for device performance and slow inference speed make it difficult to migrate complex networks to embedded and mobile devices. Although lightweight networks such as MobileNet and YOLO-LITE have greatly improved their detection speed, their accuracy still requires improvement.

## 3. Mixed YOLOv3-LITE Network

### 3.1. Mixed YOLOv3-LITE Network Structure

**Figure 2:**
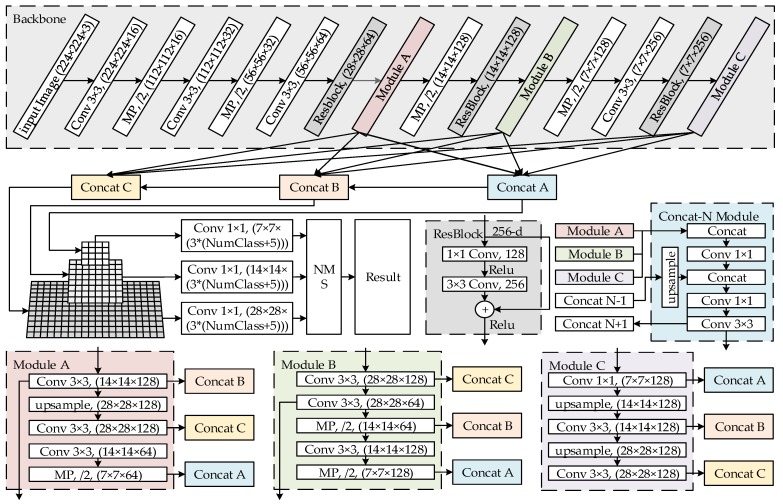
Mixed YOLOv3-LITE network structure.

To apply real-time object detection using convolutional networks on embedded platforms, such as augmented reality, we propose a simplified model structure, Mixed YOLOv3-LITE, which is a lightweight object detection framework suitable for non-GPU based devices or mobile terminals. Its simplified model structure is presented in Figure 2. The model is composed of fifteen 3 × 3 convolution layers, seven 1 × 1 convolution layers, three ResBlocks, and eight max-pooling (MP) layers. It has the following characteristics:

1. For the feature extraction part, ResBlocks and the parallel high-to-low resolution subnetworks of HRNet are added based on the backbone network of YOLO-LITE, and the shallow and deep features are deeply integrated to maintain the high-resolution features of the input image. This improves the detection precision. This part includes four 3 × 3 standard convolution layers, four maximum pooling layers, three residual blocks, modules A, B, and C for reconstructing a multi-resolution pyramid, and concat modules A, B, and C. The concat-N module is located between the backbone network and the detector, and is used to reconstruct feature maps with the same resolution at different depths.
2. For the detection part, a structure similar to that of YOLOv3 is used to reduce the number of convolution layers and channels. The detector detects the recombined feature maps of each concat-N module separately to improve the accuracy of detecting of small objects, and then selects the best detection result through maximum value suppression.

### 3.2. Mixed YOLOv3-LITE Network Module

The excellent performance of YOLOv3 is largely attributable to the application of the backbone network Darknet-53 [14]. To further improve the detection speed of the network, Mixed YOLOv3-LITE uses the shallow backbone network of YOLO-LITE to replace Darknet-53 and adds a residual structure and parallel high-to-low-resolution subnetworks to achieve the fusion of shallow and deep features, thereby improving the detection precision.

#### 3.2.1. Shallow Network and Narrow Channel

YOLO-LITE employs a backbone network with seven convolution layers and four MP layers [19]. As shown in Table 1, it is a “shallow network and narrow channel” network. The amount of computation and the number of parameters are essentially reduced in comparison with a deep network, and the detection speed of the network is improved significantly. In Mixed YOLOv3-LITE, we used a backbone network with a structure similar to that shown in Table 1, and we simultaneously narrowed the channel according to the structure at the detection end to reduce the number of parameters and amount of computation, and to improve the network training speed.

#### 3.2.2. ResBlock and Parallel High-to-Low Resolution Subnetworks

By adding a shortcut [11] to the network, the residual structure can solve the problem of the precision of the model decreasing rather than increasing when the number of layers in the VGG [27] network increases. The residual structure used in this study is consistent with the residual structure of YOLOv3 [14].

**Figure 3:**
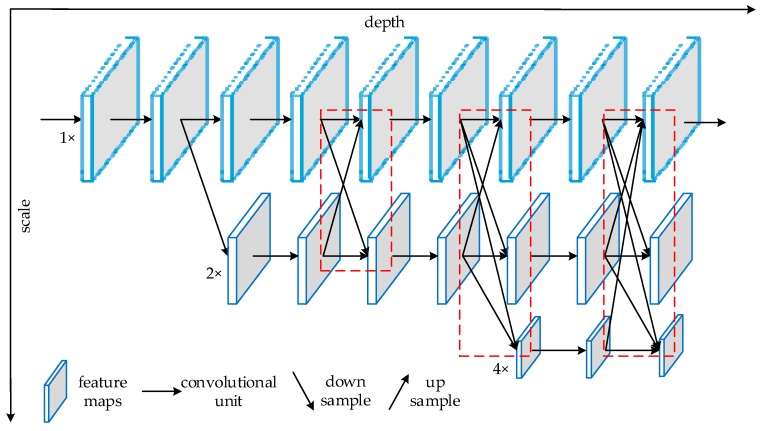
Schematic illustration of the architecture of the high-resolution network (HRNet).

The principle of parallel high-to-low-resolution subnetworks [15] is shown in Figure 3; the dotted frames are the parallel high-to-low-resolution subnetworks structure. We borrowed this idea for this study, thus the resolution of three feature images with different scales was reconstructed, fused, and then output to the detection end for object detection, thereby improving the detection precision of the network.

The residual structure and parallel high- to low-resolution subnetworks are designed to solve the degradation problem of deep networks. The difference is that the residual structure continuously transmits shallow features to deep layers through a shortcut over a small range, whereas parallel high- to low-resolution subnetworks conduct multi-resolution reconstruction of deep and shallow features at multiple scales through large- and multi-scale fusion, so that multi-scale feature maps have both deep and shallow features at the same time.

## 4. Experiment and Discussion

This section describes the experimental environment, datasets, parameter settings of the training network, and the evaluation index of the model effect. The settings of the network structure are also presented through a series of comparative experiments conducted in the process of designing the proposed network and selecting the network that yielded the optimal performance. This selection was based on a comparison of the experimental results obtained using the PASCAL VOC dataset [28]. The precision index of the network for object detection was verified using the VisDrone 2018-Det dataset [29] and the ShipData dataset. Finally, the speed index of the network was verified on the embedded platform Jetson AGX Xavier [30].

### 4.1. Experimental Details

#### 4.1.1. Experimental Environment Setup

We performed training using a TensorFlow-based version of YOLOv3 as the baseline, in which the YOLO-LITE model file [19] was also converted into the TensorFlow version for performance evaluation. The training was performed on a server equipped with an Intel Core i7 mur9700K central processing unit (CPU) and an NVIDIA RTX 2080Ti GPU. During the test, the GPU of the server was disabled, and only the CPU was used to execute the video detection script under the TensorFlow framework. The configuration details of the server are listed in Table 2. In addition, the NVIDIA Jetson AGX Xavier was used as an embedded mobile terminal for performance testing. The Jetson AGX Xavier hardware was configured as an NVIDIA self-developed eight-core ARM v8.2 64-bit CPU, a 512-core Volta GPU, and a 16-GB RAM. It is a small, fully functional low-power computing system with a module size no more than 105 mm × 105 mm, designed especially for robotic, industrial automation, and other neural network application platforms. When deployed for use with intelligent devices such as unmanned vehicles and robots, a power consumption of only 10 to 30 W can provide powerful and efficient artificial intelligence (AI), computer vision, and high-performance computing power [30,31,32].

#### 4.1.2. Experimental Datasets

The datasets used in our experiments were PASCAL VOC [28], VisDrone 2018-Det [29], and a ship dataset of remote-sensing images, which we collected from Google Earth. The PASCAL VOC [28] and VisDrone 2018-Det datasets [29] were each divided into a training set and a test set (Table 3) such that our model could be trained under the same experimental settings and compared with the benchmark model. The following is a detailed description of the three datasets.

A. PASCAL VOC

The PASCAL VOC dataset [28] is a public object detection dataset consisting of 20 categories of objects. These 20 categories are divided into four main categories: *Person*: person; *Animal*: bird, cat, cow, dog, horse, and sheep; *Vehicle*: airplane, bicycle, boat, bus, car, motorbike, and train; and *Indoor*: bottle, chair, dining table, potted plant, sofa, and tv/monitor. In the experiment, a mixed dataset composed of PASCAL VOC 2007 and 2012 was used for training and testing. The training set consisted of 16,511 images, and the test set consisted of 4592 images. Each image contained one or more object that belonged to one or more of the 20 categories used.

B. VisDrone2018-Det

VisDrone2018-Det [29] is a large UAV-based dataset consisting of 8599 images, 6471 of which were used for training, 1580 for validation, and 1580 for testing. The dataset contains rich annotations, including object bounding boxes, object categories, occlusions, and truncation rates. The label data of the training set and validation set have been made public and were used as the training set and test set, respectively. There are several real-world scenarios in the data. These datasets contain various scenes (thousands of cities and kilometers) and various weather and light conditions. We mainly focused on the following object categories in the detection of objects: pedestrians, people, cars, trucks, buses, bicycles, awning tricycles, and tricycles.

C. ShipData

The ShipData produced in this study is a remote-sensing image ship dataset with 1009 images collected by Google Earth and labeled in PASCAL VOC format. The backgrounds of the images vary greatly, and there are many different types of ships. The dataset was randomly divided into subset A (706 images) and subset B (303 images)—according to the proportion 7:3. Two subsets of data were used in the experiment. In the first round, subset A was used for training and subset B was used for testing. In the second round, subset B was used for training and subset A was used for testing.

#### 4.1.3. Evaluation Metrics

In this study, the precision, recall rate, F1 score, and mAP were used to evaluate the detection accuracy of the model. Floating point operations (FLOPs), the number of parameters, and the model size were used to evaluate the performance of the model, which was finally reflected in the frames per second (FPS) index.

The objects considered can be divided into four categories based on their actual and predicted categories [33]: true positive (TP), fault positive (FP), true negative (TN), and fault negative (FN). The relationships are shown in Table 4.

**Figure 4:**
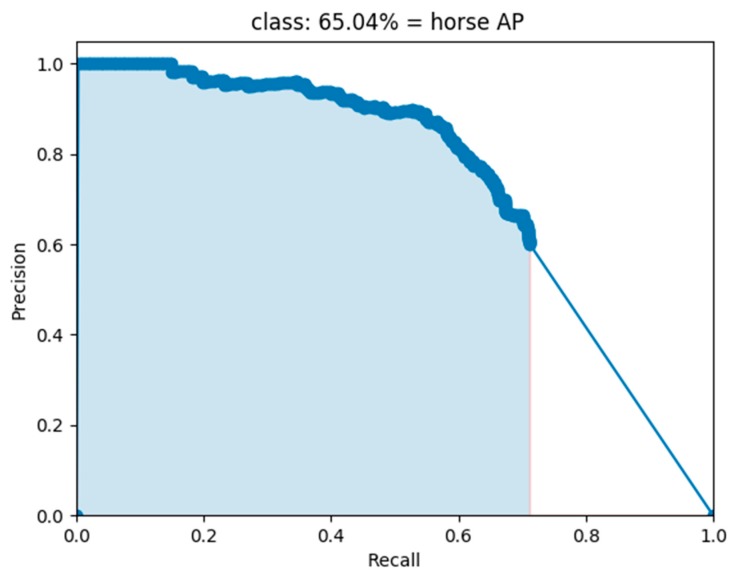
Mixed YOLOv3-LITE P-R curve of horse category in PASCAL VOC dataset.

The precision reflects the proportion of real positive examples in the positive cases determined by the classifier while the recall rate reflects the proportion of correct positive cases among the total number of positive cases. F1 is the weighted harmonic average of precision and recall, which combines the results for precision and recall. When F1 is higher, it indicates that the test method is more effective. Average precision (AP) is the area under the precision–recall (P–R) curve. For example, Figure 4 shows the P–R curve for the method in the horse category in the PASCAL VOC dataset. The AP of the horse category is the area of the shaded part in the figure, which accounts for 65.04% of the area. In general, the better the classifier, the higher the AP value. The mAP is the average of the AP value in multiple categories. The calculation method is as follows:

(1) $$precision=\frac{TP}{TP+FP}$$

(2) $$recall=\frac{TP}{TP+FN}$$

(3) $$F1=\frac{2}{1/precision+1/recall}$$

FLOP is the number of operations of the model, which can be used to evaluate the time complexity of the model. The number of parameters of the model consists of two parts: the total number of parameters and the size of the output feature graph of each layer, which can be used to evaluate the space complexity of the algorithm and the model. The overall time and space complexities of the CNN can be calculated as follows:

(4) $$Time\;\sim \;O\left(\sum_{l=1}^{D}M_{l}^{2}\cdot K_{l}^{2}\cdot C_{l-1}\cdot C_{l}\right)$$

(5) $$Space\;\sim \;O\left(\sum_{l=1}^{D}K_{l}^{2}\cdot C_{l-1}\cdot C_{l}+\sum_{l=1}^{D}M_{l}^{2}\cdot C_{l}\right)$$

In Equations (4) and (5), D represents the number of layers of the CNN, i.e., the depth of the network; l represents the lth convolution layer of the CNN; $M_{l}$ represents the side length of the output feature map for the lth convolution layer; K represents the side length of each convolution kernel; $C_{l-1}$ represents the number of input channels of the lth convolution layer, i.e., the number of output channels of the (l−1)th convolution layer; and $C_{l}$ represents the number of output channels of the lth convolution layer, i.e., the number of convolution kernels of this layer.

#### 4.1.4. Experimental Setup

This section describes the network models proposed during the design of Mixed YOLOv3-LITE and the network structure of each trial, as shown in Table 5. In all trials, 60 epochs of training were carried out using the PASCAL VOC 2007-2012 training dataset to obtain the final model. The input image size used in model training and testing was set to 224 × 224, which is consistent with that of YOLO-LITE. As YOLOv3 did not publish the precision data associated with the PASCAL VOC dataset, 60 epochs of training with YOLOv3 were performed under the same experimental environment and parameter settings, which were adopted as the evaluation baseline. The experimental results for all the networks—i.e., YOLO-LITE, YOLOv3, MobileNetV1-YOLOv3, and MobileNetV2-YOLOv3—are shown in Table 6. The details of the experiment are presented below.

A. Depthwise-separable convolutions

**Figure 5:**
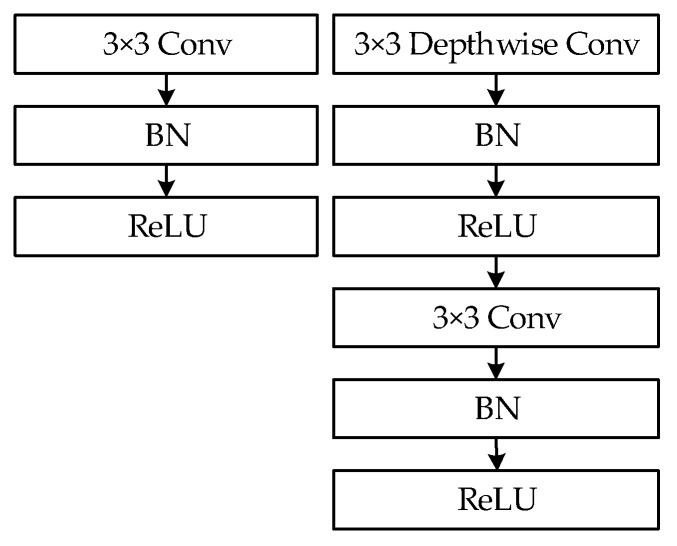
(**Left**): standard convolutional layer with batchnorm (BN) and rectified linear unit (ReLU). (**Right**): depthwise-separable convolutions with depthwise and pointwise layers, followed by batchnorm and ReLU.

Depthwise-separable convolution (as shown on the right in Figure 5), which was used in MobileNets [16] instead of ordinary convolution (as shown on the left in Figure 5), can significantly reduce the number of parameters and the amount of computation required.

By comparing YOLOv3 for Trials 1, 2, 3, 6, and 7, it is observed that without changing the network structure and by replacing ordinary convolution with deep separable convolution, the performance of the model decreases remarkably as the number of parameters, the amount of computation, and the model size increase.

B. Shallow network

In Trial 8, Darknet53 of the YOLOv3 backbone network was replaced by the seven-layer structure of the YOLO-LITE backbone network, and the number of channels in each layer was adjusted to couple with YOLOv3. Compared to the original YOLOv3, the number of parameters, amount of computation, and model size were reduced by approximately 50%. However, the mAP, recall rate, and F1 score of the model only decreased slightly. Thus, the relative efficiency of the YOLO-LITE layer backbone network structure in the lightweight model was verified.

In Trials 9 and 11, the number of computations and model size were greatly reduced in comparison to those of Trial 8 as the number of convolution layers in the detection part was gradually reduced. Meanwhile, the mAP, recall rate, and F1 score of the model decreased slightly. Thus, it was confirmed that the smaller detection part was effective for the lightweight model.

C. Narrow channel

The backbone network of YOLO-LITE is shown in Table 1. The number of channels of each layer was significantly reduced compared to that of YOLOv3. Trials 10 and 11 were designed to verify the effectiveness of the narrow-channel backbone network.

In the comparative trials between Trials 10 and 9 and between Trials 12 and 11, the number of convolution layers of the model was exactly the same, but the number of channels of Trials 10 and 11 was remarkably reduced compared to those of Trials 9 and 12. In addition, the mAP of Trials 10 and 11 decreased by 6.76% and 5.99%, respectively, and the amount of computation, the number of parameters, and the model size were reduced by factors of approximately 3.5, 7.6, and 7.7, respectively before and after adjustment. This confirmed the relative efficiency of the narrow channel in the lightweight model.

D. ResBlock

Based on Trial 12, one layer of ResBlock was added before the output of the three-scale feature maps in Trial 13, and the mAP, recall rate, and F1 score of the model increased by approximately 0.8%. However, the amount of computation, the number of parameters, and the model size were reduced by approximately 30%, 66%, and 61%, respectively, which is not cost-effective at all.

E. Parallel high- to low-resolution subnetworks

In the first set of comparative experiments, parallel high-to low-resolution subnetworks based on Trial 2 were added and the three-scale feature maps were fused before output for Trials 4 and 5. All the convolutional layers in the first set of comparative experiments were depthwise-separable convolutions. The difference between Trials 4 and 5 is that the multi-scale feature maps after the resolution reconstruction in Trial 4 are connected using the convolution operation for channel feature fusion, which made its mAP decrease by 0.24% compared with Trial 2. On the basis of Trial 4, Trial 5 reduced the number of residual blocks in the backbone, and its mAP decreased by 2.53% compared with Trial 4. It can be seen from the comparison that the parallel structure using deep separable convolution cannot improve the accuracy index of the network. Furthermore, it also reflects the number of residual blocks, that is, the depth of the backbone, which affects the network performance.

In the second set of comparative experiments, parallel high- to low-resolution subnetworks based on Trial 12 were added and the three-scale feature maps were fused before output for Trials 14 and 15. All the convolutional layers in the second set of comparative experiments were standard convolutions. The difference between Trials 14 and 15 is that the downsampling of Trial 14 was achieved by convolution with a step size of two, whereas that of Trial 15 was achieved by maximum pooling. The mAP of Trials 14 and 15 was improved by 0.05% and 1.85%, respectively. By this comparison, the effectiveness of using standard convolutions, the maximum pool, and parallel structure was demonstrated.

F. Comprehensive tests

Trials 16, 17, and 18 were all modified based on the results of Trial 15. In Trial 16, the convolution kernel of parallel high- to low-resolution subnetworks was replaced from 1 × 1 to 3 × 3. In Trial 17, one layer of ResBlock was added for each before the output of the three-scale feature map. Further, three layers of ResBlocks with inverted-bottleneck structures [17] were added for each in Trial 18 before the output of the three-scale feature map. A comparison of the results of Trials 16, 17, and 18 with those of Trial 15 shows that the mAP increased by 1.12%, 3.32%, and 2.88%, respectively. Trial 17 exhibited the best performance in terms of precision, recall rate, and F1 scores, which increased by 13.68%, 8.48%, and 12.67%, respectively, and the amount of computation of the model increased by 0.389 GFLOPs.

Trial 19 added a 3 × 3 convolution layer before the output of the parallel-structure three-scale feature map of Trial 18, and Trial 20 adjusted the number of ResBlocks of each part to three layers, based on the results of Trial 17. Trial 21 moved forward the position of the last part of the ResBlock of Trial 20 to reduce the number of channels. From the results, we can see that the mAP of Trial 19 and Trial 20 was slightly higher than that of the original network, but the amount of computation and the number of parameters increased more significantly. The operation involved in Trial 21 significantly reduced the amount of computation but sacrificed 1.31% of the mAP.

### 4.2. Experimental Results

#### 4.2.1. PASCAL VOC

**Figure 6:**
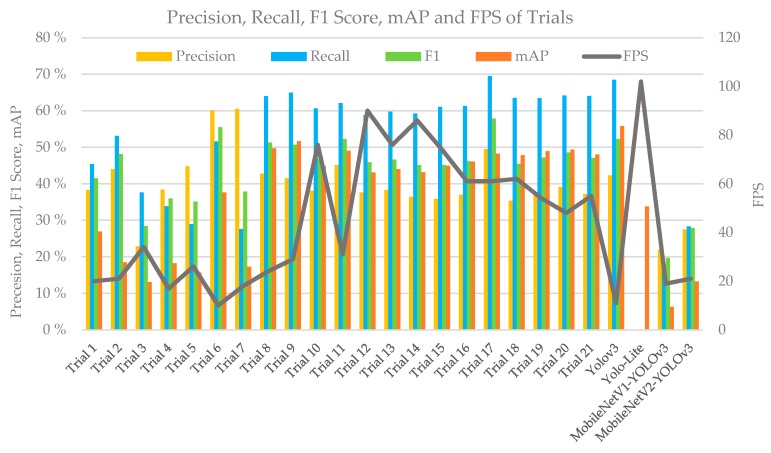
Comparison of the effects of different trials using the PASCAL VOC 2007 dataset during the construction of Mixed YOLO3-LITE.

**Figure 7:**
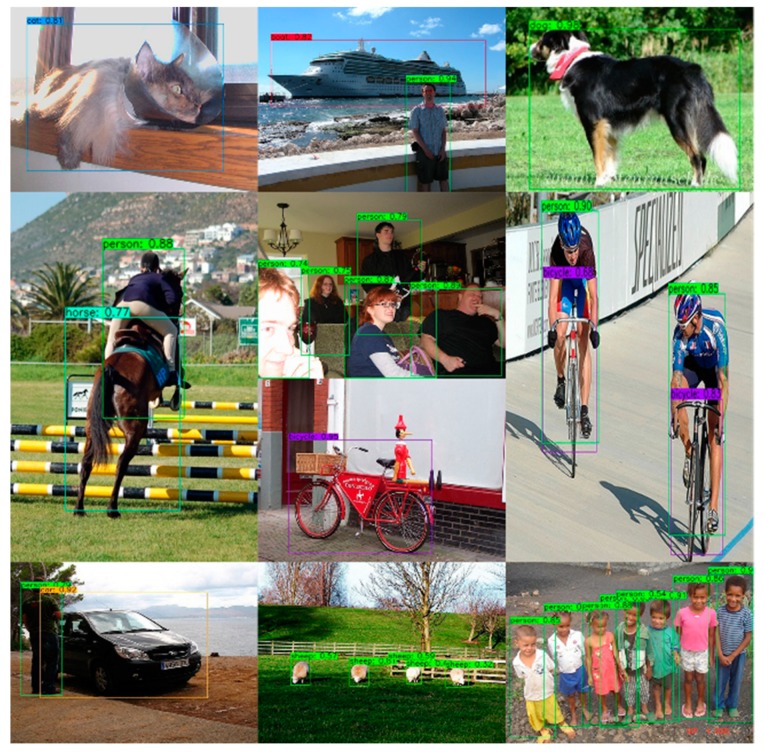
Partial test results of Mixed YOLOv3-LITE using the PASCAL VOC 2007 test set.

A total of 21 different trials were performed in this study; the results are shown in Table 6. The precision, recall rate, F1 score, mAP, and FPS of YOLO-LITE, YOLOv3, MobileNetV1-YOLOv3, MobileNetV2-YOLOv3, and the different trials obtained using the PASCAL VOC 2007 test dataset are illustrated in Figure 6. As seen from the experimental results, YOLO-LITE achieved 102 FPS (non-GPU) in the experimental environment with a high speed. However, its mAP was only 33.77%. The mAP of YOLOv3 was 55.81%, but its speed was only approximately 11 FPS (non-GPU), which is lower than that of YOLO-LITE. Based on the same parameter settings in the experimental environment, MobileNetV1-YOLOv3′s mAP is approximately 6.27% and the detection speed is approximately 19 FPS, whereas MobileNetV2-YOLOv3’s mAP is 13.26% and the detection speed is 21 FPS. These results demonstrate that it is difficult to achieve real-time object detection with non-GPU-based computers or mobile terminals. Considering the precision, recall rate, and F1 score together, Trial 17 yielded the best performance (Mixed YOLOv3-LITE) by achieving 49.53%, 69.54%, and 57.85% for the above indices, respectively. The amount of computation of the model was 2.48 GFLOPs, which is only 13% of that of YOLOv3. The model size was 20.5 MB, which is only 8.3% of that of YOLOv3, and 60 FPS was achieved in the non-GPU based experimental environment, which is approximately 5.5 times that of YOLOv3. Meanwhile, when the speed was relatively slow, the mAP was 14.48% higher than that of YOLO-LITE. A portion of the experimental results for the Mixed YOLOv3-LITE model using the PASCAL VOC 2007 testing dataset is shown in Figure 7.

#### 4.2.2. VisDrone 2018

We selected Trial 17 (i.e., Mixed YOLOv3-LITE), which yielded the best results using the PASCAL VOC dataset, to train on the VisDrone 2018 dataset. Sixty epochs of training were performed using the training set with input image data of size 832 × 832, tested using the validation dataset, and compared with the data for SlimYOLOv3 [34]. The results are shown in Table 7. The mAP of Mixed YOLOv3-LITE was clearly higher than those of the tiny-YOLOv3 and SlimYOLOv3 series networks, and it exceeded the performance of the other two networks in terms of the evaluation index of the amount of computation and model size. Mixed YOLOv3-LITE achieved 47 FPS in the test environment when an NVIDIA RTX 2080Ti GPU was used.

**Figure 8:**
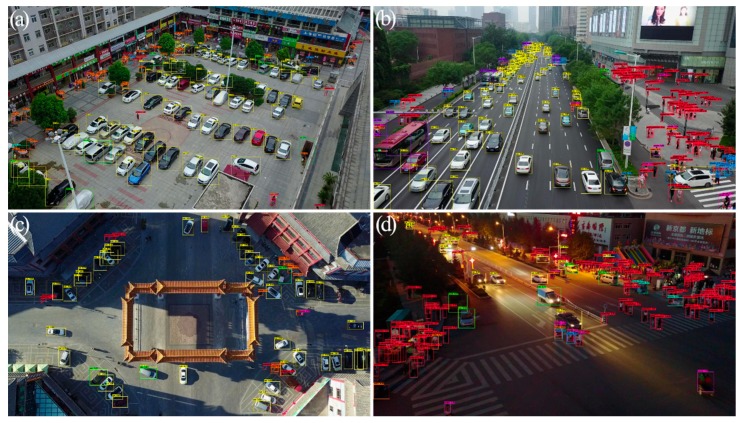
Partial test results obtained with Mixed YOLOv3-LITE using VisDrone2018-Det Val dataset. (**a**) Static object image, (**b**) dynamic object image, (**c**) orthographic image, and (**d**) bad light image.

The detection efficacy of Mixed YOLOv3-LITE (832 × 832) for each type of object using the VisDrone2018-Det validation dataset is shown in Table 8. The data category distribution of the VisDrone2018-Det dataset is highly uneven, which is more challenging. For example, instances of cars as objects accounted for approximately 36.29% of the total instances, whereas awning tricycles accounted for relatively few sample objects, precisely only 1.37% of the total number of instances. This introduces problems of imbalance to the detector optimization. The AP achieved for cars was 70.79%, whereas that for awning tricycles it was only 6.24%. In the Mixed YOLOv3-LITE design process, the convolution layer structure was reorganized and deleted, but the problem of category imbalance was not dealt with, which provides guidance for further optimization of the network in the future. A portion of the results for Mixed YOLOv3-LITE obtained using the VisDrone2018-Det validation dataset is shown in Figure 8.

#### 4.2.3. ShipData Results

**Figure 9:**
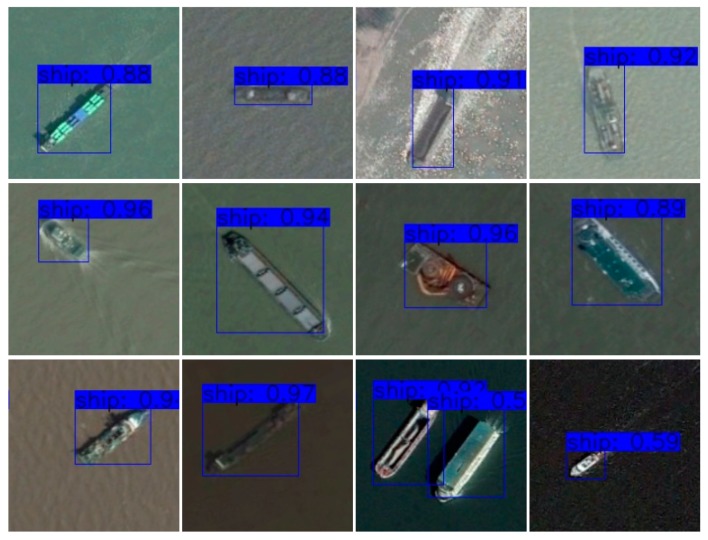
Partial test results for Mixed YOLOv3-LITE for ShipData.

Mixed YOLOv3-LITE and YOLOv3 were trained using the ShipData dataset. The experiment was divided into two parts: (1) training using subset A and testing using subset B; (2) training using subset B and testing using subset A. The input image size was 224 × 224, other training and test parameter values were the same, and 60 epochs of training were conducted. The results are shown in Table 9. The results for a single category dataset show that when the proportion of the training data was 70%, the mAPs of Mixed YOLOv3-LITE and YOLOv3 were 98.88% and 98.60%, respectively. When the proportion of training data was 30%, the recall rates of mAP of Mixed YOLOv3-LITE and YOLOv3 were 64.68% and 51.65%, respectively. However, the precision and F1 scores were slightly lower. The results for the two groups of experiments show that the network proposed in this study yields better detection results for a single category of the remote-sensing image ShipData set. A portion of the detection results for Mixed YOLOv3-LITE in the first experiment is shown in Figure 9.

#### 4.2.4. Performance Tests Based on Embedded Platform

Mixed YOLOv3-LITE was tested with a Jetson AGX Xavier device; the results are shown in Table 10. When inputting an image with a size of 224 × 224, it reached 43 FPS. When used in UAV imaging with an adjusted image size of 832 × 832, it still reached 13 FPS. In summary, the proposed method can meet the real-time requirements established.

## 5. Conclusions

In this study, we proposed an efficient lightweight object detection network that uses a shallow-layer, narrow-channel, and multi-scale feature image parallel fusion structure. On the one hand, the residual block and the parallel structure fuse the deep and shallow features and output multi-scale feature maps to maximize the utilization of the original features to improve the accuracy rate. On the other hand, the detector is constructed using shallower and narrower convolutional layers than YOLOv3, so as to reduce the amount of calculation and the number of trainable parameters and speed up the network operation. Thus, we proposed Mixed YOLOv3-LITE, which has a narrower and shallower structure than that of YOLOv3. Our proposed method has fewer trainable parameters, thereby significantly reducing the amount of computation and increasing running speed. Compared to YOLO-LITE, the detection precision is greatly improved. Computing power and power consumption are generally limited with non-GPU-based devices, mobile terminals, and all types of intelligent devices; thus, efficient lightweight-depth neural networks are needed to ensure a longer battery life for all types of devices and make them work stably. After comprehensive consideration, Mixed YOLOv3-LITE has been proven capable of achieving higher efficiency and better performance than YOLOv3 and YOLO-LITE on mobile terminals and other devices.

## Figures and Tables

**Table 1:** YOLO-LITE architecture.

Layer	Filters	Size	Stride
C1	16	3 × 3	1
MP		2 × 2	2
C2	32	3 × 3	1
MP		2 × 2	2
C3	64	3 × 3	1
MP		2 × 2	2
C4	128	3 × 3	1
MP		2 × 2	2
C5	128	3 × 3	1
MP		2 × 2	2
C6	256	3 × 3	1
C7	125	1×1	1
The region			

**Table 2:** Hardware environment configuration information.

		Operating System	Memory	CPU	Video Card
Training environment		Ubuntu 16.04	48 GB	Intel Core i7-9700k	GeForce RTX 2080Ti
Testing environment	With GPU	Ubuntu 16.04	48 GB	Intel Core i7-9700k	GeForce RTX 2080Ti
	Without GPU	Ubuntu 16.04	48 GB	Intel Core i7-9700k	without

**Table 3:** Overview of PASCAL VOC and VisDrone datasets.

Dataset	Training Images	Test Images	Number of Classes
PASCAL VOC 2007 & 2012	16,511	4952	20
VisDrone 2018-Det	6471	548	10
ShipData	706(Subset A)	303 (Subset B)	1

**Table 4:** Confusion matrix.

	Predicted	1	0	Total
Actual				
1		True Positive (TP)	False Negative (FN)	Actual Positive (TP + FN)
0		False Positive (FP)	True Negative (TN)	Actual Negative (FP + TN)
Total		Predicted Positive (TP + FP)	Predicted Negative (FN + TN)	TP + FN + FP + TN

**Table 5:** Network structure description of different trials.

Model	Structure Description
YOLO-LITE	YOLO-LITE raw network structure [19], as shown in Table 1
YOLOv3	YOLOv3 raw network structure [14], as shown in Figure 1
MobileNetV1-YOLOv3	Backbone uses MobileNetV1 while using YOLOv3 detector part
MobileNetV2-YOLOv3	Backbone uses MobileNetV2 while using YOLOv3 detector part
Trial 1	All convolution layers in YOLOv3 were replaced by depth-separable convolution, and the number of ResBlocks in Darknet53 was replaced from 1-2-8-8-4 to 1-2-4-6-4.
Trial 2	The convolution layer was reduced in the detector part of Trial 1 by one layer.
Trial 3	The number of ResBlocks in the backbone network of Trial 2 was reduced from 1-2-4-6-4 to 1-1-1-1-1.
Trial 4	A parallel structure was added based on Trial 2, the resolution was reconstructed using a 1 × 1 convolutional kernel, and the channel was fused using a 3 × 3 convolutional kernel after the connection.
Trial 5	Based on Trial 4, the number of ResBlocks in the backbone network was replaced by 1-1-2-4-2, and the resolution was reconstructed using a 3 × 3 convolutional kernel.
Trial 6	A parallel structure was added based on YOLOv3, which used a 1 × 1 ordinary convolution.
Trial 7	All convolutions in Trial 6 were replaced by depth-separable convolutions.
Trial 8	The region was exactly the same as that of YOLOv3, and the last layer became wider when the backbone extracted features.
Trial 9	The backbone was exactly the same as that in Trial 8, and three region levels were reduced by two layers for each.
Trial 10	Three region levels were reduced by two layers for each, the region was narrowed simultaneously, and the backbone was exactly the same as that of YOLO-LITE.
Trial 11	The backbone was exactly the same as that in Trial 8, and three region levels were reduced by four layers for each.
Trial 12	The backbone was exactly the same as that of YOLO-LITE, three region levels were reduced by four layers for each, and the region was narrowed simultaneously (three region levels were reduced by two layers for each based on Trial 10).
Trial 13	Three ResBlocks were added based on Trial 12.
Trial 14	Three HR structures were added based on Trial 12.
Trial 15	Based on Trial 14, the downsampling method was changed from the convolution step to the maximum pool, and a layer of convolution was added after the downsampling.
Trial 16	The convolution kernel of the last layer of HR was changed from 1 × 1 to 3 × 3 based on Trial 15.
Trial 17	Three ResBlocks were added to Trial 15.
Trial 18	Nine layers of inverted-bottleneck ResBlocks were added to Trial 15.
Trial 19	Based on Trial 18, the output layers of HR structure were increased by one 3 × 3 convolution layer for each, for a total of three layers.
Trial 20	The number of ResBlocks per part was adjusted to three, based on Trial 17.
Trial 21	The last ResBlocks was moved forward to reduce the number of channels, based on Trial 20.

**Table 6:** Results of different trials using PASCAL VOC dataset.

Model	Layers		Model Size (MB)	GFLOPs	Params (M)	FPS	mAP	Precision	Recall	F1
	Backbone	The Region								
**YOLO-LITE**	7	5	2.3	0.482	not reported	102	33.77	not reported	not reported	not reported
**YOLOv3**	52	23	246.9	19.098	61.626	11	55.81	42.29	68.48	52.29
**MobileNetV1-YOLOv3**	27	23	97.1	6.234	24.246	19	6.27	21.95	17.90	19.72
**MobileNetV2-YOLOv3**	53	23	93.5	5.622	23.270	21	13.26	27.48	28.34	27.90
**Trial 1**	43	23	20.8	2.142	5.136	20	26.87	38.27	45.4	41.53
**Trial 2**	43	17	15.9	1.854	3.911	21	18.46	44	53.11	48.12
**Trial 3**	19	11	7.7	1.091	1.908	34	13.01	22.86	37.65	28.45
**Trial 4**	49	17	39.6	3.496	9.83	17	18.22	38.43	33.84	35.99
**Trial 5**	34	17	35.1	2.832	8.714	26	15.69	44.77	28.91	35.13
**Trial 6**	58	23	270.3	20.702	67.467	10	37.61	60.09	51.58	55.51
**Trial 7**	58	23	76.3	5.737	19	18	17.29	60.53	27.58	37.89
**Trial 8**	7	23	136.8	7.892	34.151	24	49.73	42.8	64.02	51.3
**Trial 9**	7	17	109.2	6.337	27.265	29	51.69	41.53	64.95	50.67
**Trial 10**	7	17	14.3	1.975	3.555	76	44.93	38.09	60.66	46.8
**Trial 11**	7	11	81.6	4.782	20.378	31	49.07	45.12	62.08	52.26
**Trial 12**	7	11	10.4	1.299	2.66	90	43.08	37.63	58.78	45.88
**Trial 13**	13	11	17.3	1.688	4.293	76	43.99	38.26	59.72	46.64
**Trial 14**	13	11	11	1.401	2.727	86	43.13	36.38	59.28	45.09
**Trial 15**	16	11	13.6	2.091	3.366	74	44.93	35.85	61.06	45.18
**Trial 16**	16	11	18.8	2.896	4.669	61	46.05	37.02	61.33	46.17
**Trial 17**	23	11	20.5	2.480	5.089	61	48.25	49.53	69.54	57.85
**Trial 18**	34	11	18	2.229	4.464	62	47.81	35.36	63.54	45.43
**Trial 19**	37	11	22.1	2.935	5.483	54	48.94	37.52	63.45	47.15
**Trial 20**	34	11	34.3	2.867	5.582	48	49.32	39.09	64.15	48.58
**Trial 21**	34	11	21.7	2.808	5.387	55	48.01	37.17	64.03	47.03

**Table 7:** Evaluation results for baseline models and Mixed YOLOv3-LITE for VisDrone 2018-Det.

Model Name	Precision (%)	Recall (%)	F1 (%)	mAP (%)	GFLOPs	Model Size (MB)	FPS
**Mixed YOLOv3-LITE**	39.19	37.80	37.99	**28.50**	**2.48**	20.5	47
tiny-YOLOv3	23.40	20.10	21.00	11.00	21.82	33.1	52
YOLOv3-spp1	42.90	36.70	39.20	25.50	262.84	239	15
YOLOv3-spp3	43.50	38.00	40.20	26.40	284.10	243	14
SlimYOLOv3-spp3-50	45.90	36.00	39.80	25.80	122	79.6	23
SlimYOLOv3-spp3-90	36.90	33.80	34.00	23.90	39.89	30.6	24
SlimYOLOv3-spp3-95	36.10	31.60	32.20	21.20	26.29	19.4	28

Note: Tiny-YOLOv3 and SlimYOLOv3 series network FPS data were measured in the NVIDIA GTX1080Ti environment used in Reference [34].

**Table 8:** Detection performance of Mixed YOLOv3-LITE (832 × 832) for each category using VisDrone2018-Det validation dataset.

Class	Images	Instances	Precision (%)	Recall (%)	F1 (%)	mAP (%)
**awning-tricycle**	**548**	**532**	**26.12**	**13.16**	**17.50**	**6.24**
bicycle	548	1287	20.08	19.35	19.71	7.92
bus	548	251	47.60	47.41	47.50	40.87
**car**	**548**	**14,064**	**61.36**	**76.54**	**68.11**	**70.79**
motor	548	5125	44.08	43.61	43.85	32.74
pedestrian	548	8844	34.57	45.66	39.35	34.50
people	548	4886	42.32	35.45	38.58	23.39
tricycle	548	1045	33.38	24.69	28.38	15.25
truck	548	750	33.81	31.47	32.60	21.94
van	548	1975	48.55	40.71	44.29	31.31
overall	548	38,759	39.19	37.80	37.99	28.50

**Table 9:** Evaluation results for YOLOv3 and Mixed YOLOv3-LITE for ShipData.

Train Dataset	Test Dataset	Model Name	Precision (%)	Recall (%)	F1 (%)	mAP (%)
Subset A	Subset B	YOLOv3	98.83	98.68	97.24	98.60
		**Mixed YOLOv3-LITE**	**96.15**	**99.01**	**97.56**	**98.88**
Subset B	Subset A	YOLOv3	58.79	62.04	60.37	51.65
		Mixed YOLOv3-LITE	31.03	83.29	45.21	64.68

**Table 10:** Comparison of experimental results for different network architectures.

Model	Input Size	FPS
Mixed YOLOv3-LITE	224 × 224	43
Mixed YOLOv3-LITE	832 × 832	13
